# Treatment of Chylothorax complicating pulmonary resection with hypertonic glucose Pleurodesis

**DOI:** 10.1186/s13019-021-01462-6

**Published:** 2021-05-28

**Authors:** Kejian Zhang, Changyuan Li, Mingrui Zhang, Yang Li

**Affiliations:** 1 Department of Thoracic Surgery, Jilin Cancer Hospital, Chang Chun, Jilin 130021 People’s Republic of China; 2 Department of Thoracic Surgery, First Hospital of Jilin University, Xinmin street 71, Chang Chun, 130021 People’s Republic of China; 3 Hospital of Stomatology, Jilin University, Chang Chun, 130021 People’s Republic of China

**Keywords:** Chylothorax; hypertonic glucose, Pneumonectomy, Pleurodesis

## Abstract

**Background:**

To retrospectively assess the efficacy of hypertonic glucose pleurodesis for treatment of chylothorax after pulmonary resection.

**Methods:**

Out of a total of 8252 patients who underwent pulmonary resection (at least lobectomy) at department of thoracic surgery, between June 2008 and December 2015, 58 patients (0.7%) developed postoperative chylothorax. All patients received conservative treatment, including thoracic closed drainage, oral fasting, and total parenteral nutrition.

**Results:**

Conservative treatment was successful in 50 (86.2%) patients, while eight patients [mean age: 58.0 years (range, 45–75)] were treated with hypertonic glucose pleurodesis. All eight patients had undergone operation for lung cancer (four squamous cell carcinomas and four adenocarcinomas). The bronchial stump was covered by pleural flap in three patients. After pleurodesis, three patients developed fever but without empyema; thoracentesis was performed in two patients. The mean time interval between pleurodesis and operation was 4.3 days (range,3–5) days. The average length of stay was 23.1 days (range, 18–31). No recurrent pleural effusion was observed over a mean follow-up duration of 28 months.

**Conclusion:**

Hypertonic glucose pleurodesis performed via the chest drainage tube is a viable treatment option for chylothorax after lung resection, prior to resorting to a thoracoscopic or thoracotomic ductus thoracicus ligation of the thoracic duct leak. It is a simple, safe and efficient modality associated with rapid recovery and less pain.

## Background

Chylothorax is an uncommon complication of pulmonary resection with an incidence rate at 0.25–2.0% [1, 2] . The condition is associated with a high mortality rate of up to 50% in untreated patients. Conservative management includes chest tube drainage, low-fat diet, oral fasting combined with total parenteral nutrition (TPN). The optimal method of chylothorax treatment remains controversial. Though thoracoscopic ductus thoracicus ligation showed effective results, the timing it is unclear about the operation opportunity to convert from conservative treatment to surgery.

In this study, we describe the use of hypertonic glucose pleurodesis through the chest drainage tube in eight cases of chylothorax, which resulted in complete and rapid recovery. Previous reports on use of chemical agent pleurodesis [3, 4] suggest that inflammatory response induced by hypertonic glucose may generate extensive adhesions in the wall and prevent chylous fluid effusion. We intended to explore its impact on chylothorax after pulmonary resection.

### Patients and methods

A total of 8252 patients underwent pulmonary resection (at least lobectomy) at our department of thoracic surgerybetween June 2008 and December 2015. A total of 58 patients (0.7%) developed postoperative chylothorax. Diagnostic criteria used for chylothorax was drain triglyceride level > 110 mg/dL or lymphocytes > 90%. All patients were initially treated with thoracic close drainage, oral fasting, and TPN. Conservative treatment was successful in 50 (86.2%) patients, including eight patients (0.7%) were treated with hypertonic glucose pleurodesis. Except for the conservative treatment patients, the other cases received video-assisted thoracoscopic surgery (VATS) or thoracotomy.

### Hypertonic glucose pleurodesis

We injected 200 mL 50% hypertonic glucose into the thoracic cavity through the chest tube. The procedure was performed at bedside under aseptic conditions. After injection, the chest tube was clamped for 2 h. The patient was made to assume different positions during this time period to facilitate distribution of hypertonic glucose in the pleural cavity. Chest radiography was reviewed after 1 day to check for potential encapsulated effusion. No thoracentesis was needed unless dyspnea or fever. The chest tube was removed when the drainage volume reduced to < 50 mL Oral intake was started 2 days later. Chest radiography was checked before discharge.

## Results

All eight patients treated with hypertonic glucose pleurodesis were operated for lung carcinoma. Of these, four patients had squamous cell carcinoma and four had adenocarcinoma. Mean age of patients was 58.0 years (range, 45–75). The baseline clinical characteristics of these patients are summarized in Table 1 including age, sex, histology, TNM-stage, lobectomy type, time interval between pleurodesis and operation, length of stay, fever, thoracentesis, and bronchial stump coverage.

**Table 1:** Relevant data on patients treated with hypertonic glucose pleurodesis

Patient No	Gender	Age in years	Histology	Stage	Lobectomy	Drainage volume in the 24 h prior to pleurodesis (in mL)	Days after operation pleurodesis was performed	Total hospital stay in days	Fever	Thoracentesis	Stump covered with pleural flap
1	M	55	Adenocarcinoma	T2N1 (IIB)	Right upper	1050	5	21	No	No	Yes
2	F	75	Adenocarcinoma	T1N2 (IIIA)	Right upper	650	3	24	No	No	No
3	M	52	Squamous carcinoma	T1N2 (IIIA)	Right middle and lower	460	4	19	Yes	Yes	Yes
4	M	64	Squamous carcinoma	T1N1 (IIA)	Right upper	480	5	22	No	No	No
5	F	45	Squamous carcinoma	T2N0 (IB)	Right middle and lower	780	5	28	Yes	No	Yes
6	M	56	Adenocarcinoma	T2N1 (IIB)	Right lower	850	4	31	Yes	Yes	No
7	M	62	Adenocarcinoma	T2N0 (IB)	Right upper	680	3	18	No	No	No
8	M	55	Squamous carcinoma	T2N0(IB)	Right upper	500	5	22	No	No	No

The bronchial stump was covered by pleura in three patients. After pleurodesis, three patients developed fever but without empyema; of these thoracocentesis were performed in two patients. The mean time interval between pleurodesis and operation was 4.3 days (range, 3–5). The average length of stay in hospital was 23.1 days (range, 18–31). None of the patients showed recurrent pleural effusion over a mean follow-up duration of 28 months.

**Fig. 1:**
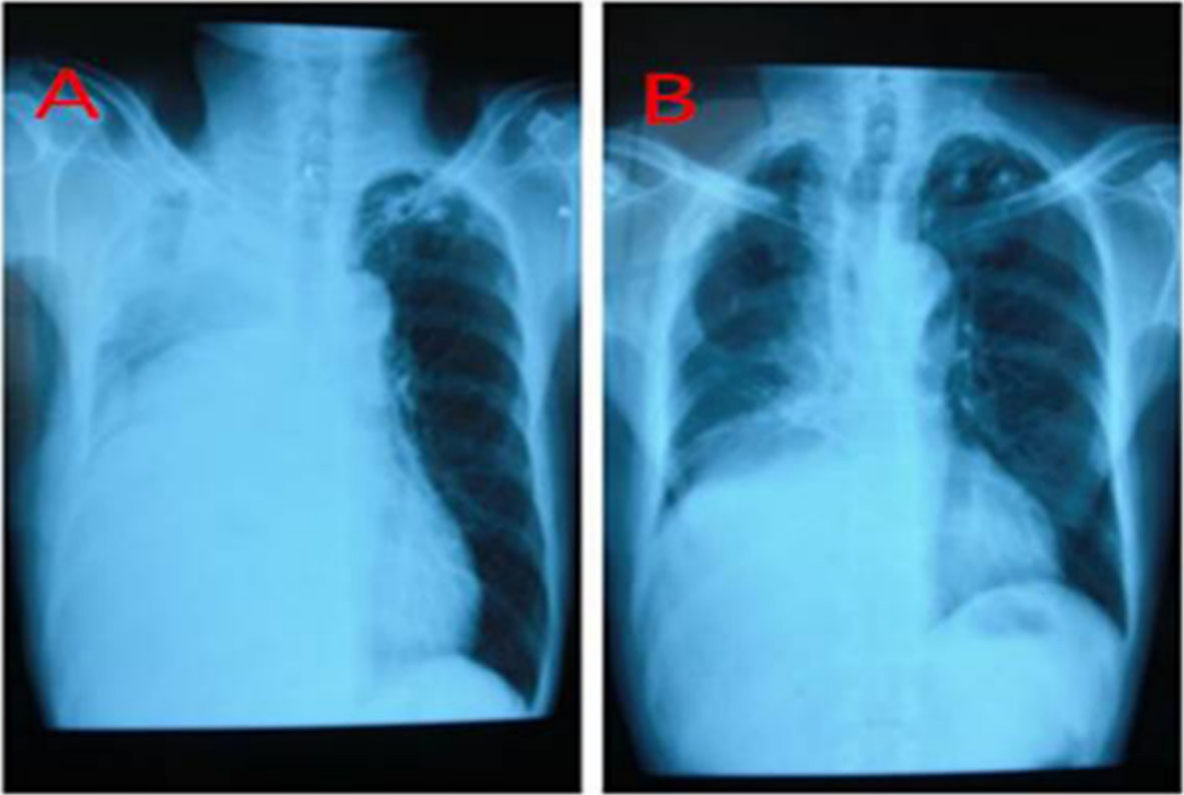
**a**. Chest X-ray of patient 1 after removal of chest drainage tube showing some accumulation of fluid in the right upper paramediastinal zone; (**b**). The chest radiography of the same patient two months later showed no signs of intrathoracic fluid accumulation or high density zones

In one case, the patient received right upper lobectomy with systematic lymph node dissection. A total of 1300 mL chylaceous fluid was drained during the first 24 h after the surgery. The daily drainage volume was in excess of 1000 mL in the following 3 days. Hypertonic glucose pleurodesis was performed on the fifth day after surgery. No drainage discharge occurred in the following 2 days; therefore, the chest tube was removed. Chest radiography revealed high density shadow in right upper paramediastinal zone (Fig. 1a). The patient did not complain of dyspnea or fever. Oral feeding was started on the tenth day after operation and the patient discharged on the 17th day after operation. No high density shadow was observed on two-month follow-up (Fig. 1b).

## Discussion

Aggressive mediastinal lymph node dissection and incomplete lymphatic ligation may lead to chylothorax. Treatment options include observation, chest drainage, oral fasting, low fat diet with or without medium chain triglycerides supplementation, administration of somatostatin and analogs, pleurodesis using biogel or talcum powder, pleural-peritoneal cavity bypass, percutaneous catheterization thoracic duct embolization, and prophylactic thoracic duct ligation [5–8]. Conservative therapy is often effective. The optimal method of treatment remains controversial as variable results have been obtained from conservative and operative treatment. Several studies have demonstrated the efficacy of VATS in chylous fistula [9–13]. The specific indications for VATS vs. thoracotomy are not clear.

Chylothorax commonly occurs after esophagectomy, which may damage the thoracic duct. Chylothorax after lobectomy is relatively rare and the leak usually appears at the level of the thoracic duct branch. Most patients can be handled through conservative treatment. In a retrospective analysis by Cerfolio et al. [14], 89% of patients with post esophagectomy chylothorax required reoperation as compared to only 38% of those with post-lobectomy chylothorax. However, chylothorax occurring after pneumonectomy usually requires reoperation [15]. Use of hypertonic glucose pleurodesis may help increase the success rate of conservative management.

Numerous reasons may lead to chylothorax after surgery, of which surgical procedure review is extremely important. Mediastinal lymph node dissection is certainly the main cause for the occurrence of chylothorax after pulmonary resection [15, 16]. We retrospectively analyzed the possible reasons that caused chylothorax in our eight cases. During pulmonary resection, all patients underwent radical mediastinal lymph node dissection for lung cancer surgery. In such cases, dissection of lymph node IV and VII is liable to damage the branch of the thoracic duct. Additionally, surgeons should be wary of the possibility of creating a chylous leak by elevation of a pleural flap in the mediastinum. For instance, the right upper or intermediate bronchial stump was covered with the pleural flap under the azygos vein. A branch of the main duct is also liable o injury during pleural flap isolation. Conservative treatment succeeded in these eight cases, as their formation was the damage of the branch of the thoracic duct instead of itself. On literature search, we did not find any reports of similar management of chylothorax after pulmonary resection.

Although chemical pleurodesis involves an inherent risk of creating a multiloculated chylothorax, it is liable to spontaneous absorption. Encapsulated pleural effusion was observed in right upper paramediastinal zone after hypertonic. No encapsulated pleural effusion or high density shadow was observed after discharge.

Injection of hypertonic glucose through the chest tube may potentially contaminate chest tube and pleural space. However, no empyema occurred in these eight cases because of antibacterial properties of chylous fluid [17]. Even so, it is worth emphasizing the importance of sterile operation and povidone iodine disinfection. In addition, repeated manipulation may increase the risk of bacterial contamination. Therefore, we suggest that hypertonic glucose pleurodesis should be performed on no more than two occasions, and as early as possible to limit the risk of empyema. We performed hypertonic glucose pleurodesis within 3–5 days of the daily chest tube drainage volume in excess of 400 mL.

The clinical significance and advantage of hypertonic glucose pleurodesis in treating chylothorax after pulmonary resection was to avoid reoperation, reduce the duration of hospital stay, and decrease the hospitalization costs. Pleurodesis can lead to pleural adhesion and obliteration of chylous leaks. Use of hypertonic glucose avoided the multiple side effects associated with use of other reagents, such as allergic sensitization, chest pain, impairment of thyroid function, local cytotoxic mucosal and skin lesions, and generalized edema [18]. Moreover, it allows injection of a large volume to generate adhesion throughout the thoracic cavity [19, 20]. All of the eight patients treated by hypertonic glucose pleurodesis showed no other complications. Wider use of hypertonic glucose pleurodesis to treat chylothorax after pulmonary resection is recommended.

## Conclusions

We verified that hypertonic glucose pleurodesis performed through the chest drainage tube is an effective treatment for chylothorax and should be tried prior to thoracoscopic or thoracotomic thoracic duct ligation. It is a simple, safe, less painful and efficient treatment that allows for a rapid recovery.

## Data Availability

The datasets used and/or analysed during the current study are available from the corresponding author on reasonable request.

## Abbreviations

TPI: Total parenteral nutrition
VATS: Video-assisted thoracoscopic surgery
TNM: (T:primary tumor, N:regional lymph nodes, M: distant metastasis)
